# Contrast Analysis on Spin Transport of Multi-Periodic Exotic States in the XXZ Chain

**DOI:** 10.3390/e27101070

**Published:** 2025-10-15

**Authors:** Shixian Jiang, Jianpeng Liu, Yongqiang Li

**Affiliations:** 1 College of Science, National University of Defense Technology, Changsha 410073, China; 2 Hunan Key Laboratory of Extreme Matter and Applications, National University of Defense Technology, Changsha 410073, China; 3 Hunan Research Center of the Basic Discipline for Physical States, National University of Defense Technology, Changsha 410073, China

**Keywords:** nonequilibrium dynamics, quantum XXZ model, quantum spin transport, dynamical scalings

## Abstract

Quantum spin transport in integrable systems reveals a rich nonequilibrium phenomena that challenges the conventional hydrodynamic framework. Recent advances in ultracold atom experiments with state preparation and single-site addressing have enabled the understanding of this anomalous behavior. Particularly, the full universality characterization of exotic initial states, as well as their measurement representation, remain unknown. By employing tensor network and contrast methods, we systematically investigate spin transport in the quantum XXZ spin chain and extract dynamical scaling exponents emerging from two paradigmatic and experimentally attainable initial states, i.e., multi-periodic domain-wall (MPDW) and spin-helix (SH) states. Our results using different values of anisotropic parameters Δ∈[0,1.2] demonstrate the evident impeded transport and the difference between the two states with increasing Δ values. Large-scale and consistent simulations confirm the contrast method as a viable scaling extraction approach for exotic states with periodicity within experimentally accessible timescales. Our work establishes a foundation for studying initial memory and the corresponding relations of emergent transport behavior in nonequilibrium quantum systems, opening avenues for the identification of their unique universality classes.

## 1. Introduction

Quantum transport in nonequilibrium many-body systems is a central research frontier in physics. Prototypical studies focus on the dynamics of globally conserved quantities, such as charge, energy density, and spin current [1,2,3,4,5]. These studies have provided valuable insights into the behavior of quantum spin transport. Remarkably, scaling invariance reveals that the properties of different systems exhibit identical behavior despite distinct microscopic details, which is related to the concept of emergent universality [6,7,8,9,10,11,12,13,14,15,16]. While significant progress has been achieved in characterizing spin transport in various models [17,18,19,20,21,22,23,24], a comprehensive understanding of the scaling laws of quantum spin transport remains unexplored. A critical gap persists in the comprehensive understanding of the role of the initial-state condition in nonequilibrium quantum statistics. Recent experimental advances have fundamentally reshaped this research field. Platforms such as ultracold atoms in optical lattices combined with quantum gas microscopy [25,26] and superconducting quantum processors [27] provide single-site resolution and feasible product-state generation, offering unprecedented access to the dynamics of quantum many-body systems.

In integrable systems, generalized hydrodynamics (GHD) provides a foundational framework predicting ballistic spin transport, which is characterized by the dynamical exponent z=1 [4,7,28,29,30,31]. However, intriguing initial-state dependence has been revealed in theoretical studies [7,28]. For the paradigmatic domain-wall (DW) initial state, spin transport quantified by the transferred current through the center point exhibits anomalous superdiffusion at the anisotropic parameter Δ=1, which is reminiscent of Kardar–Parisi–Zhang (KPZ) universality with z=3/2 [25]. On the other hand, recent experiments show that the spin-helix (SH) state exhibits normal diffusion with z=2 [32,33,34].

The Bethe Ansatz provides an exact framework for the XXZ model’s equilibrium properties and certain dynamical correlations, yet its application to the real-time dynamics of spatially inhomogeneous states remains computationally challenging [7,27,35,36,37].

Advanced simulations based on the matrix product operator (MPO) [38,39,40,41,42,43] and the adaptive GHD method [4,7,10,28,29,30,41,44,45,46] therefore serve as a powerful tool for a broad range of spin transport phenomena from low to infinite temperature. Moreover, the established framework holds promise for exploring transport in open quantum systems, including integrable models with dissipation and measurement back-action [39]. Although ballistic fluctuation theory has established the detailed understanding in ballistic regimes, the relationships are largely unknown in the other two regimes displaying strong initial-state dependence [47,48]. It is recognized that the proximity of the multi-periodic SH initial state to an eigenstate leads to nearly frozen transport, making direct current measurements used in the DW state likely impossible and beyond experimental timescales [32,33,34]. Hence, the universal scaling and dynamical exponent obtained from the contrast of SH states are expected to capture the same physics as the transferred current in the DW state. However, to our knowledge, comprehensive studies and detailed simulations on this subject remain scarce. Specifically, the characterization method and diverse dynamical responses of such exotic states under highly nonequilibrium processes are still not well understood.

In this work, we address these questions concerning quantum transport in the paradigmatic quantum XXZ spin chain. We use the framework based on the contrast function to characterize spin transport emerging from multi-periodic exotic states across distinct regimes. By extending the concept of the single-periodic DW state to the multi-periodic domain-wall (MPDW) state, we are able to simultaneously extract the scaling exponents ν, which correspond to the inverse of the dynamical exponents. Using a unified contrast method, our large-scale simulations reveal that these two states exhibit distinct universal behaviors with different scaling exponents. Within the single-fermion-particle framework, the distinct symmetries of the two states indicate that their evolution is governed by different protective symmetries.

This universal scaling reconciles the apparent discrepancy between MPDW and SH initial states as well as different anisotropic parameters, manifesting the complexity of nonequilibrium dynamics.

The structure of this article is organized as follows. In Section 2, we introduce the one-dimensional XXZ spin model and two types of initial states, namely the MPDW state and the SH state. We also describe the time-dependent variational principle (TDVP) algorithm based on tensor networks used for numerical simulations.

In Section 3, we analyze the spin transport properties. We begin by examining the single-fermion-particle information of the initial states via the Jordan–Wigner transformation and introduce the contrast method as a tool for extracting the scaling exponents. Comprehensive convergence tests are performed to validate the behavior of the normalized contrast. We then systematically investigate the dependence of the scaling exponents on the anisotropic parameter for both initial states, which is accompanied by a detailed analysis of the contrast dynamics. Data collapse is further employed to confirm the scaling behavior. Finally, the article concludes in Section 4 with a summary of the main findings and an outlook on potential future research directions.

## 2. Theoretical Framework and Numerical Methods

### 2.1. One-Dimensional XXZ Spin Model

We consider the one-dimensional spin-1/2 XXZ model with nearest-neighbor interactions and open boundary condition, which is described by the Hamiltonian

(1) $$\hat{H}=-J\sum_{i=1}^{L-1}\left({\hat{S}}_{i}^{x}{\hat{S}}_{i+1}^{x}+{\hat{S}}_{i}^{y}{\hat{S}}_{i+1}^{y}+\Delta {\hat{S}}_{i}^{z}{\hat{S}}_{i+1}^{z}\right),$$

where J=1 sets the coupling strength, ${\hat{S}}_{i}^{x,y,z}$ represents spin-1/2 operators at site *i*, *L* denotes the system size, and Δ is the anisotropy parameter. The Hamiltonian possesses U(1) symmetry, which is reflected by the commutation relation $[\hat{H},{\hat{S}}_{\mathrm{tot}}^{z}]=0$ with ${\hat{S}}_{\mathrm{tot}}^{z}=\sum_{i=1}^{L}{\hat{S}}_{i}^{z}$. This symmetry corresponds to invariance under global spin rotations about the *z*-axis, implying a globally conserved quantity and serving as a foundation for constructing hydrodynamic descriptions. The symmetry properties vary distinctly with the anisotropy parameter Δ. Note that we only consider positive values in this paper.

1. Easy-plane regime Δ∈[0,1): The system has U(1) symmetry. Geometrically, this corresponds to rotational invariance exclusively around the *z*-axis in spin space.
2. Isotropic point Δ=1: The system has SU(2) symmetry. This higher symmetry implies invariance under arbitrary global rotations in spin space, which is analogous to the full rotational symmetry of a sphere. It signifies the conservation of all components of the total spin.
3. Easy-axis regime Δ∈(1,+∞): The system has Z(2) symmetry, which breaks rotational invariance in the x-y plane and exhibits spatial inversion symmetry along the z-axis.

Note that the XX model of Δ=0 can be mapped to the free-fermion system and exhibits ballistic transport via the Jordan–Wigner transformation [49]. We pay particular attention to these three regimes, where spin transport exhibits pronounced initial-state dependence.

### 2.2. Initial States and Emergent Nonequilibrium Dynamics

It was reported that quantum transport in spin models behaves as strongly initial-state dependence, which is beyond a conventional hydrodynamic description [7,28]. Here, we focus on two experimentally accessible initial states, the MPDW state, extending from the conventional DW state [12,25,50], and the SH [32,33,34] state, which gives rise to distinct spin transport behavior.

**Figure 1:**
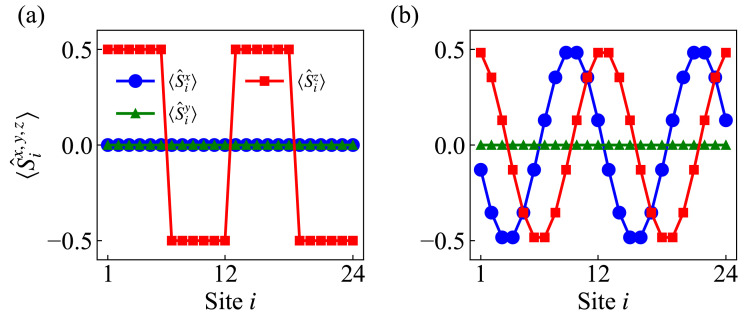
Real-space spin distributions for (**a**) the MPDW state and (**b**) the SH state for a system size of L=24 and wavelength λ=12. The blue squares, green triangles and red circle represent the expectation values $\langle {\hat{S}}_{i}^{x}\rangle$, $\langle {\hat{S}}_{i}^{y}\rangle$, and $\langle {\hat{S}}_{i}^{z}\rangle$, respectively.

The MPDW state is a product state combined with a periodic domain-wall structure

(2) $$|\varphi_{\mathrm{MPDW}}\rangle =\overset{L/\lambda }{\underset{k=1}{⨂}}\left({|↑\rangle }^{⊗\lambda /2}⊗{|↓\rangle }^{⊗\lambda /2}\right).$$

where λ denotes the integer period length of the domain-wall structure. The total length *L* is selected as an integer multiple of the period, expressed as $L=n_{p}\lambda$, where $n_{p}\in Z$ represents the number of periods. This MPDW state retains the characteristic domain-wall structure within each individual period of fully polarized up and down local spins on the left and right parts, respectively (see illustration of system L=24 and λ=12 in Figure 1a). It is reported that the spin transport of a single-periodic DW state exhibits anomalous superdiffusion with a universal profile [16,51]:

(3) $$\langle S^{z}\left(x,t\right)\rangle ∼f_{\mathrm{KPZ}}\left(x/t^{1/z}\right),$$

where the dynamical exponent z=3/2 obeys KPZ universality [25,27], and $f_{\mathrm{KPZ}}$ denotes the KPZ scaling function. Although superdiffusion dominates over intermediate timescales, a number of researchers have argued that there is an eventual recovery of normal diffusion at a very late time [52,53,54,55,56].

On the other hand, the SH state is a spatially winding state defined as [32,34]

(4) $$|\varphi_{\mathrm{SH}}\rangle =\overset{L}{\underset{i=1}{⨂}}\left[\cos\left(Qi/2\right){|↑\rangle }_{i}-\sin\left(Qi/2\right){|↓\rangle }_{i}\right],$$

where the wavevector Q=2π/λ and *i* denotes the lattice site index. An example of its real-space spin distribution for system size L=24 and wavelength λ=12 is shown in Figure 1b. The spin transport of the SH initial state displays markedly different behavior. Within experimentally accessible timescales, it follows diffusive dynamics characterized by a dynamical exponent of z≈2 [32,33,34]. Above all, these two states serve as paradigmatic examples to study the state-dependent transport behavior, and the question is raised of how initial conditions can influence dynamical modes within the same XXZ model.

### 2.3. TDVP Algorithm Based on Tensor Network

To overcome the exponential wall problem in quantum many-body simulations [57], we employ tensor network methods that provide compressed representations of quantum states. For a spin-1/2 system of size *L*, the Hilbert space dimension scales as $2^{L}$, making exact diagonalization infeasible for large systems. The matrix product state (MPS) formalism enables an efficient representation of quantum states by expressing the many-body wave function as a chain of tensors:

(5) $$|\Psi \rangle =\sum_{\sigma_{1},\ldots ,\sigma_{L}}A_{1}^{\sigma_{1}}A_{2}^{\sigma_{2}}\ldots A_{L}^{\sigma_{L}}|\sigma_{1}\sigma_{2}\ldots \sigma_{L}\rangle ,$$

where $A_{i}$ represents site tensors with physical indices $\sigma_{i}$ and auxiliary bond indices connecting neighboring sites. Singular value decomposition (SVD) provides the mathematical foundation for tensor network compression. For any matrix $M\in C^{m\times n}$, the factorization $M=USV^{†}$ yields singular values that quantify entanglement entropy across system partitions. By truncating small singular values while preserving the χ largest ones, we achieve a controlled approximation where the bond dimension χ governs both accuracy and computational cost. This truncation reduces the parameter count from exponential in *L* to polynomial in χ, making large-scale simulations computationally tractable while preserving the physically relevant entanglement structure.

Tensor network-based numerical methods have demonstrated remarkable progress in studying spin transport phenomena in quantum magnets [7,40,41,42,58].

The TDVP method provides a framework for simulating time-dependent dynamics by projecting the Schrödinger equation onto a variational manifold [59]. For MPS, this projection enables long-time evolution while preserving key physical quantities like energy and norm. The standard Schrödinger equation is

(6) $$i\hbar \frac{\partial }{\partial t}|\psi \left(t\right)\rangle =\hat{H}|\psi \left(t\right)\rangle .$$

TDVP projects this equation onto the MPS manifold

(7) $$i\hbar \frac{\partial }{\partial t}|\psi \left(t\right)\rangle =P_{M}\hat{H}|\psi \left(t\right)\rangle ,$$

where $P_{M}$ is the tangent-space projector to the MPS manifold. This ensures the evolved state remains within the variational space. In practice, two primary integration schemes are employed for applying TDVP to MPS, differing in their treatment of the bond dimension χ. The one-site TDVP preserves bond dimension χ throughout the evolution. No truncation occurs and total energy as well as norm are conserved, but it would introduce projection errors due to the fixed bond dimension. The two-site TDVP allows the bond dimension to grow during evolution. Truncation becomes necessary to prevent the exponential growth of χ, which violates strict energy conservation but reduces projection errors [60,61,62].

It is important to notice the limitations of this approach. The projection error originates from restricting the full quantum dynamics to a constrained variational subspace, which cannot perfectly represent the complete time-evolved state. Furthermore, practical simulations face the challenge of entanglement growth. During time evolution, entanglement generally increases and the bond dimension of the matrix product state must grow accordingly to represent the state accurately. Although the two-site TDVP alleviates this issue by allowing the bond dimension growth, truncation becomes necessary to control computational cost. This bond dimension saturation ultimately limits the reachable time scales. Once the bond dimension reaches its preset limit, it can no longer faithfully capture the continuing growth of entanglement, and truncation errors begin to accumulate.

In our simulations, to obtain the scaling exponents of the spin transport in the long-time dynamics, we employ the TDVP method using the ITensor library [59,60,63]. Specifically, to balance finite-size effects and computational cost, a hybrid scheme is applied [60,61,62], combining the two-site and one-site TDVP methods to leverage their respective advantages. The simulation starts with the two-site TDVP to effectively capture rapid entanglement growth in the initial dynamics while adaptively increasing the bond dimension. Once the bond dimension attains a predefined threshold $\chi_{\mathrm{th}}$, the algorithm is switched to the one-site TDVP. Our convergence tests confirm that the key results presented in this work are fully converged and numerically reliable.

## 3. Dynamical Scaling of Spin Transport

### 3.1. Single-Particle Information of Initial States via Jordan–Wigner Transformation

The local spin distribution of two states in Figure 1 shows a distinct difference in the *x* component, winding pattern and detailed structure within each wavelength, despite the same periodicity. To better identify these initial conditions, we map the spin operators to fermion operators via the Jordan–Wigner transformation, which reads

(8) $$\begin{array}{cc} {\hat{\sigma }}_{j}^{+} & =2e^{-i\pi \Sigma_{k=1}^{j-1}n_{k}}{\hat{a}}_{j}^{†},\\ {\hat{\sigma }}_{j}^{-} & =2e^{i\pi \Sigma_{k=1}^{j-1}n_{k}}{\hat{a}}_{j},\\ {\hat{\sigma }}_{j}^{z} & =2{\hat{n}}_{j}-1. \end{array}$$

Here, ${\hat{a}}_{j}\;\left({\hat{a}}_{j}^{†}\right)$ represent the fermion annihilation (creation) operator, respectively. ${\hat{n}}_{j}={\hat{a}}_{j}^{†}{\hat{a}}_{j}$ is the fermion occupation number operator. ${\hat{\sigma }}_{j}^{+,-,z}$ represent the Pauli up, down and z-component operators, respectively. Next, we calculate the single-particle density matrix of the initial product state in the fermion framework

(9) $$\begin{array}{c} \rho_{ij}=\langle {\hat{a}}_{i}^{†}{\hat{a}}_{j}\rangle =\langle {\hat{\sigma }}_{i}^{+}{\hat{\sigma }}_{j}^{-}\prod_{k=i+1}^{j-1}{\hat{\sigma }}_{k}^{z}\rangle =\langle {\hat{\sigma }}_{i}^{+}\rangle \langle {\hat{\sigma }}_{j}^{-}\rangle \prod_{k=i+1}^{j-1}\langle {\hat{\sigma }}_{k}^{z}\rangle , \end{array}$$

which shows the hidden correlation and structure in off-diagonal elements [58]. Here, 〈⋯〉=〈φ|⋯|φ〉 represents an inner product. Note that the product ranges between *i* and *j*, and decomposition is applicable when initial local spins are uncorrelated. Considering two states in Equations (2) and (4), we define the uniform formation

(10) $$|\varphi \rangle =\overset{L}{\underset{k=1}{⨂}}\left(c_{k}{|↑\rangle }_{k}+d_{k}{|↓\rangle }_{k}\right)$$

where $c_{k}\;\left(d_{k}\right)$ represent the amplitude of the up (down) basis, respectively. Then, we can obtain the matrix element

(11) $$\rho_{ij}=\left\{\begin{array}{c} |c_{i}{|}^{2},\;\;\;\;i=j\\ c_{j}d_{j}^{*}d_{i}c_{i}^{*}\left[\prod_{k=i+1}^{j-1}\left({\left|d_{k}\right|}^{2}-{\left|c_{k}\right|}^{2}\right)\right],\;\;\;\;i<j\\ \rho_{ji},\;\;\;\;i>j. \end{array}\right.$$

**Figure 2:**
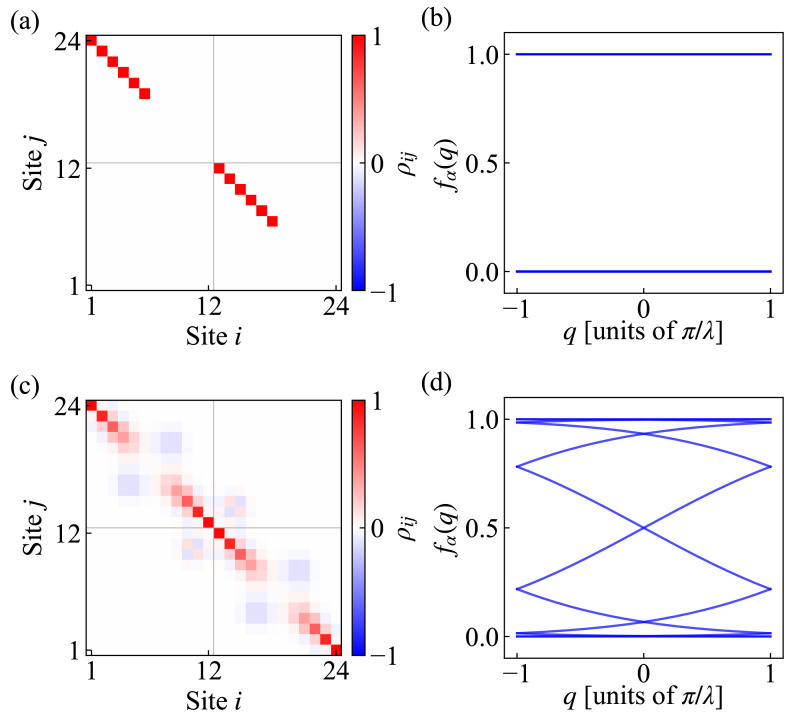
Single-particle information of two initial states with size L=24, λ=12 and open boundary condition, corresponding to states in Figure 1. Top row panels show (**a**) single-particle density matrix $\rho_{ij}$ and (**b**) occupation number bands $f_{\alpha }\left(q\right)$ for fermionic representation of the MPDW state, and bottom row panels correspondingly show (**c**) single-particle density matrix and (**d**) occupation number bands of the SH state.

We take the example of two states as shown in Figure 1 with L=24,λ=12 and present their matrix element distributions in Figure 2a,c. It can be directly seen that the MPDW state has no off-diagonal elements, as opposed to the SH state. And the SH state exhibits a decay mode of off-diagonal elements to zero when |i−j|>λ, which can be explained as a result of the product formation of z-component parts in Equation (9) [58]. It also indicates that there is exponential decay for initial correlations of different sites. In contrast, the MPDW state clearly has zero off-diagonal elements, since it only has z-component with amplitude $|\langle {\hat{\sigma }}_{i}^{z}\rangle |=1$ and obeys uncorrelated fermion particle behavior.

Then, we would like to see the difference in the spectrum structure in the fermion picture. The relation between the occupation number and single-particle density matrix shows an eigenvalue equation

(12) $$\sum_{i}\rho_{ij}v_{j}^{\alpha }=f_{\alpha }v_{j}^{\alpha }$$

where $f_{\alpha }$ is the fermion number in the α mode and $v_{j}^{\alpha }$ is the wave function. This relation can be extended to momentum space by introducing Bloch’s theorem $v_{j}^{\alpha }=e^{iqj}\phi_{j}^{\alpha }$ with periodic condition $\phi_{j+\lambda }^{\alpha }=\phi_{j}^{\alpha }$ and wavevector $q\in [-\frac{\pi }{\lambda },\;\frac{\pi }{\lambda })$. The eigenmatrix is reduced to λ×λ size [58]

(13) $$\sum_{j}\Gamma_{ij}\left(q\right)\phi_{j}^{\alpha }=f_{\alpha }\left(q\right)\phi_{i}^{\alpha }$$

with the formula

(14) $$\Gamma_{mn}\left(q\right)=\sum_{k}\rho_{m,n+k\lambda }e^{iq(n+k\lambda -m)}=\left(\rho_{mn}+\frac{\rho_{m,n+\lambda }e^{iq\lambda }}{1-\chi e^{iq\lambda }}+\frac{\rho_{m,n-\lambda }e^{-iq\lambda }}{1-\chi e^{-iq\lambda }}\right)e^{iq(m-n)},$$

where $\chi =\prod_{j=1}^{\lambda }\left({\left|d_{j}\right|}^{2}-{\left|c_{j}\right|}^{2}\right)$ and 0⩽m,n<λ. We can obtain the band structure for the occupation number f(q) at a specific wavevector *q*, as shown in Figure 2b,d. The MPDW state only has two bands of 0 occupation and 1 occupation uniformly, which corresponds to whether the fermion number is empty or filled on those local sites. It was reported that this behavior is the general behavior of classical and uncorrelated spin configurations [58]. On the other hand, the SH state has no gaps for $f_{\alpha }$ in the whole spectrum, which is clearly different from the DW state. Reflection symmetry is preserved by mapping q→−q in the x-z plane or $f_{\alpha }\to 1-f_{\alpha }$ when λ is even [58]. Above all, we can clearly see the difference in the single-particle information of two initial states, which directly leads to distinct and universal transport behavior during evolution.

### 3.2. Contrast Method and Convergence Tests

In this section, we introduce a unified scaling approach based on the contrast method for two types of multi-periodic initial states: namely, the SH state and the MPDW state. We employ the contrast function of spin polarization as a standard and experimentally viable probe, which is defined as [32]

(15) $$K\left(\lambda ,t\right)=\sum_{i=1}^{L}\cos\left(Qi+\theta \right)\langle {\hat{S}}_{i}^{z}\left(t\right)\rangle ,$$

where Q=2π/λ is the wavevector of the initial state, and θ denotes the initial phase of the *z* component. For the SH state, the phase is θ=0, while for the MPDW state, it is θ=−π/2. The contrast method is particularly suitable for probing transport in multi-periodic initial states like the SH and MPDW states. By systematically tuning the wavelength parameter λ, this approach allows us to explore spin dynamics across a broad range of length scales. This capability is important for identifying universal scaling laws in spin transport that hold irrespective of the specific number of periods in the initial state, thereby providing strong evidence for the universality class of a given state. To quantify the decay of contrast across states of different wavelengths, we normalize Equation (15) by the initial contrast, yielding the normalized contrast K(λ,t)/K(λ,0).

**Figure 3:**
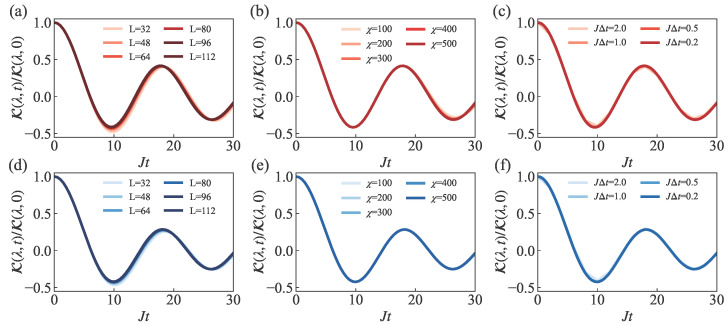
Convergence analysis of the normalized contrast K(λ,t)/K(λ,0) for wavelength λ=16 at Δ=0. (**a**–**c**) SH state and (**d**–**f**) MPDW state. (**a**,**d**) System size dependence for N=32, 48, 64, 80, 96, 112; (**b**,**e**) bond dimension dependence for χ=100,200,300,400,500; (**c**,**f**) time step dependence for JΔt=2.0,1.0,0.5,0.2. Darker colors in all subfigures correspond to more accurate parameter values.

Before presenting our conclusive data on the SH and MPDW states, we performed extensive convergence tests with respect to system size *L*, bond dimension χ, and time step JΔt to ensure the reliability of our numerical simulations, as shown in Figure 3. These tests were conducted using a representative intermediate wavelength of λ=16, which serves as a typical case study for our convergence analysis. We selected the convergence parameter as Δ=0, since at this point, the XXZ spin model reduces to the exactly solvable XX model [64]. The contrast decay in this model for the SH state has been demonstrated to follow the form of a Bessel function [42,58,65], whose periodic oscillatory behavior helps amplify the effects of parameter variations. The tests systematically examined system sizes *L* from 32 to 112, bond dimensions χ from 100 to 500, and time steps JΔt from 2.0 to 0.2. The results demonstrate robust convergence across this broad parameter range. Based on a balanced consideration of numerical accuracy and computational cost, we selected L=96, χ=500, and JΔt=0.5 for our numerical simulations. This parameter set was confirmed to yield reliable convergence in the results for the representative wavelength λ=16.

### 3.3. Anisotropic Parameter Dependence of Scaling Exponents of Two States

**Figure 4:**
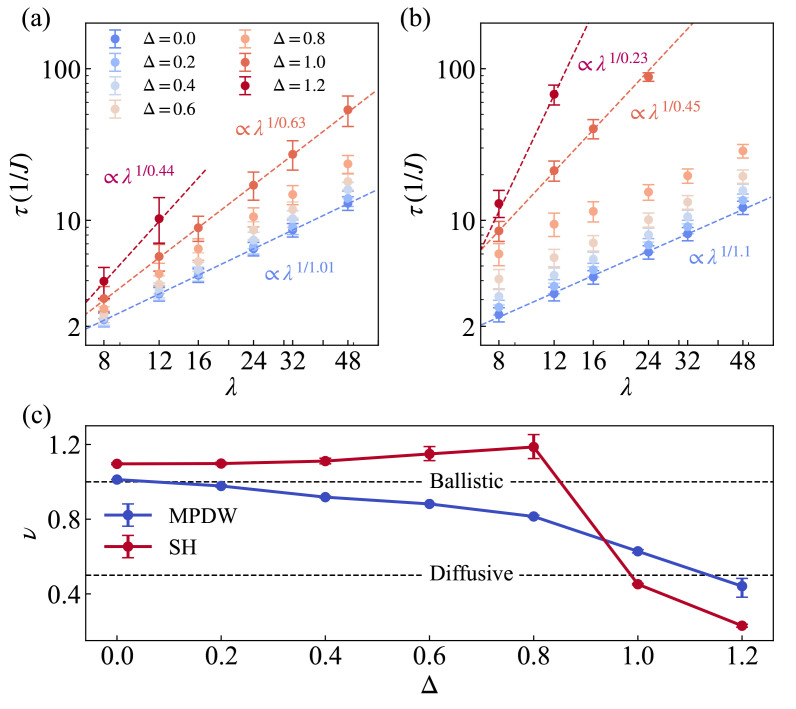
State-dependent scaling exponents of spin transport from contrast decay for (**a**) the MPDW and (**b**) the SH initial states. Results are shown for anisotropies Δ=0.0,0.2,…,1.2. The decay time τ is determined as the averaged time for the normalized contrast K(λ,t)/K(λ,0) to decay from 1 to threshold values of 0.3, 0.4, and 0.5. Error bars represent the variation in τ across these three threshold values. Dashed lines in each panel represent power-law fits $\tau \propto \lambda^{1/\nu }$ for Δ=0.0, 1.0, and 1.2, with the exponents obtained by averaging the scaling behavior observed across the three threshold measurements, yielding ν≈1.01, 0.63, 0.44 for the MPDW state and ν≈1.1, 0.45, 0.23 for the SH state. The wavelengths used for fitting, λ=8,12,16,24,32,48, are chosen such that the system size *L* is an integer multiple of λ. (**c**) Scaling exponents ν as a function of Δ, extracted from the power-law fits in (**a**,**b**), highlighting the state-dependent nature of spin transport. The error bars in (**c**) indicate the variation in ν values obtained from the three independent threshold-based measurements. Simulation parameters: system size L=96, bond dimension χ=500, time step JΔt=0.5.

Here, we first present the main results of this paper. Following the contrast method in Equation (15), we analyze the spin dynamics for both the MPDW and SH initial states in the XXZ model with Δ∈[0,1.2]. Figure 4a,b shows the scaling behavior for the MPDW and SH states, respectively, where the dashed lines indicate power-law fits. The scaling exponent ν is extracted from the relation $\tau \propto \lambda^{1/\nu }$, where τ denotes the decay time for the normalized contrast K(λ,t)/K(λ,0) to decay from 1 to a specified threshold. To assess the robustness of the scaling behavior, we employ three different threshold values of 0.3, 0.4, and 0.5, which represent moderate and experimentally realistic choices consistent with recent studies [26,32]. The final scaling exponent ν is obtained by averaging the exponents derived from these three independent procedures. The error bars represent the variation in τ across these threshold values, providing an estimate of the uncertainty in our scaling analysis. The three fitted curves correspond to key anisotropy values Δ=0.0, 1.0, and 1.2. The wavelengths used for scaling are λ=8,12,16,24,32,48. For larger Δ values and longer wavelengths, the contrast decays exceptionally slowly. When the contrast does not decay to any of the threshold values of 0.3, 0.4, and 0.5 within our evolution time of Jt=100, the corresponding wavelength data points are not included in the scaling plots in Figure 4a,b. For these two states, we can see a similar decreasing trend of scaling exponents from almost ballistic to subdiffusive regimes but with different values.

All scaling exponents obtained from Figure 4a,b are summarized in Figure 4c. In the easy-plane region with Δ∈[0,1), the transport exponents ν remain close to 1, and the SH state consistently exhibits a larger exponent than the MPDW state throughout the regime. At Δ=1, the previous pattern whereby the SH state consistently exhibited larger scaling exponents than the MPDW state is reversed, reflecting a significant change in their transport behavior. The MPDW state exhibits superdiffusive transport, which is characterized by a logarithmically enhanced diffusion with ν≈0.63. In contrast, the SH state demonstrates almost normal diffusion with ν≈0.45, which is consistent with experimental observations [32]. When Δ=1.2, the system enters the easy-axis regime, where spin transport is nearly frozen for pure states [53,56,66]. Our observed subdiffusive behavior with markedly small scaling exponents for both MPDW and SH states at this anisotropy clearly manifests these expected characteristics of suppressed transport. Overall, this dependence manifests clearly in the different scaling exponents for the two initial states in the whole range of Δ.

### 3.4. Analysis of Contrast Scaling for the SH State

**Figure 5:**
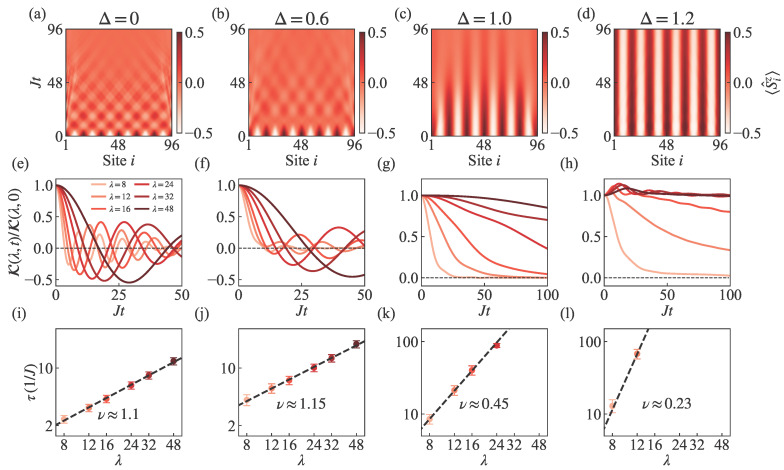
Scaling of spin transport in the SH state. Columns from left to right correspond to anisotropy values ranging from Δ=0 to Δ=1.2. (**a**–**d**) displays time evolutionmaps of real-space spin distribution for a system size L=96 with wavelength λ=16. (**e**–**h**) presents the decay of normalized contrast K(λ,t)/K(λ,0) with each panel examining six different wavelengths with λ=8,12,16,24,32,48 (from light to dark). Black dashed lines indicate the zero-contrast baseline. For Δ=1.2, the decay time τ for long wavelengths could not be determined within the simulation time range due to slow decay. (**i**–**l**) demonstrates the power-law relationship between wavelength λ and decay time τ via $\tau \propto \lambda^{1/\nu }$, where the decay time τ is measured using three different threshold values of 0.3, 0.4, and 0.5 for the normalized contrast decay. Error bars represent the variation in τ across these threshold values, and the fitted scaling exponents of ν≈1.1,1.15,0.45,0.23 for the respective anisotropic parameters are obtained by averaging the exponents derived from the three independent threshold measurements. System size L=96, time step JΔt=0.5, and bond dimension χ=500.

We now provide detailed numerical evidence to support these findings. We analyze the relaxation dynamics and spin transport scaling by examining four anisotropic parameters, Δ=0,0.6,1.0,1.2. Starting from the SH initial state, Figure 5a–d display real space–time evolution maps of spin distribution with size L=96, total time Jt=96 and wavelength λ=16. For the easy-plane cases at Δ=0 and 0.6, we observe the oscillations from spin exchange interaction, which manifests as regular patterns of crossing and flipping in the space–time distribution. These systems eventually reach a steady state through time evolution. At the isotropic point Δ=1, the flipping behavior disappears and the relaxation to a steady state becomes significantly slower. For Δ=1.2 in the easy-axis regime, spin transport appears nearly frozen, which relates to the long-lived states caused by the SH initial state being close to the eigenstate [34].

Correspondingly, Figure 5e–h show the time evolution of the normalized contrast K(λ,t)/K(λ,0), which corresponds to the degree of imbalance in the system, as seen in the spin distribution maps displayed in the top row. The easy-plane regime exhibits persistent oscillations around zero contrast. Slower relaxation is associated with larger Δ values and longer wavelengths. The isotropic point shows smooth decay without oscillatory behavior, while the easy-axis regime exhibits markedly slower decay, especially for initial states with longer periods, which largely preserve their initial state information. Black dashed lines indicate the zero-contrast reference. As a result, Figure 5i–l demonstrate the extraction of spin transport scaling exponents through the power-law relationship between wavelength λ and decay time τ, which is expressed as $\tau \propto \lambda^{1/\nu }$. The decay time τ is determined using three different threshold values of 0.3, 0.4, and 0.5 for the normalized contrast decay, and the scaling exponents are obtained by averaging the exponents derived from these three independent measurements. The scaling exponents obtained for Δ=0,0.6,1.0,1.2 are ν≈1.1,1.15,0.45,0.23, respectively. We observe a dramatic change in the scaling exponent at the isotropic point Δ=1, where transport changes from ballistic dynamics with ν≈1 in the easy-plane regime to diffusive dynamics with ν≈0.5 in the easy-axis regime.

### 3.5. Analysis of Contrast Scaling for the MPDW State

**Figure 6:**
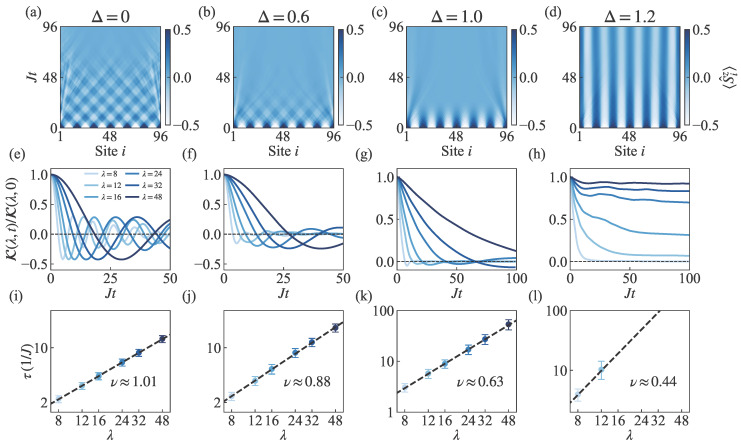
Scaling of spin transport in the MPDW state, using the same presentation format as Figure 5. Columns correspond to anisotropies from Δ=0 to Δ=1.2. (**a**–**d**) displays time evolution maps of real-space spin distribution for a system size L=96 with wavelength λ=16. (**e**–**h**) presents the decay of normalized contrast K(λ,t)/K(λ,0) with each panel examining six different wavelengths with λ=8,12,16,24,32,48 (from light to dark). Black dashed lines indicate the zero-contrast baseline. For Δ=1.2, the decay time τ for long wavelengths could not be determined within the simulation time range due to slow decay. (**i**–**l**) displays scaling exponents ν≈1.01,0.88,0.63,0.44 obtained from the power-law fit $\tau \propto \lambda^{1/\nu }$. Similar challenges were encountered in determining τ for long wavelengths at Δ=1.2 due to slow decay. All other simulation parameters remain identical with system size L=96, time step JΔt=0.5, and bond dimension χ=500.

Figure 6 provides detailed information on spin transport scaling for the MPDW state. Similarly, the spin distribution evolution maps shown in Figure 6a–d exhibit crossing and flipping patterns in the easy-plane regime, decay patterns at the isotropic point, and almost frozen evolution in the easy-axis regime. These trends are consistent with the SH state. Figure 6e–h show that in the easy-plane regime, the normalized contrast oscillates around zero with faster decay corresponding to more pronounced oscillations. It is noteworthy that the contrast decays more rapidly here compared with the SH state at the isotropic point, reaching near zero within the interval Jt<50. In the easy-axis regime, the contrast function always decays very slowly despite different initial states. Figure 6i–l yields scaling exponents of ν≈1.01,0.88,0.63,0.44 for Δ=0,0.6,1.0,1.2, respectively, illustrating the transition of spin transport from ballistic to subdiffusive regimes across the range Δ∈[0,1.2].

As shown in Figure 6d, the MPDW initial state with λ=16 at Δ=1.2 exhibits an absence of transport, manifesting as a dynamical freezing of the spin polarization that lies outside the emergent hydrodynamic regime. Consequently, the data point for λ=16 is excluded from the scaling analysis in Figure 6l. This ensures that the scaling exponents for the MPDW state are extracted from a consistent fitting window as those for the SH state. In summary, for the regime 0≤Δ≤1, the analysis includes all wavelengths λ=8,12,16,24,32,48 for which the normalized contrast decays to at least one of the defined thresholds within the simulated time window of Jt=100. For the easy-plane point Δ=1.2, only the shorter wavelengths λ=8,12 are used to prevent misleading effects from the dynamical freezing observed at longer wavelengths. All fitting results are presented in Figure 4.

These results quantitatively demonstrate the differences in spin transport between the MPDW and SH states. In particular, at the isotropic point Δ=1, the MPDW state exhibits superdiffusive transport, whereas the SH state shows diffusive transport, highlighting the pronounced initial-state dependence of spin transport at this critical point.

### 3.6. Data Collapse of Scaling Behavior

**Figure 7:**
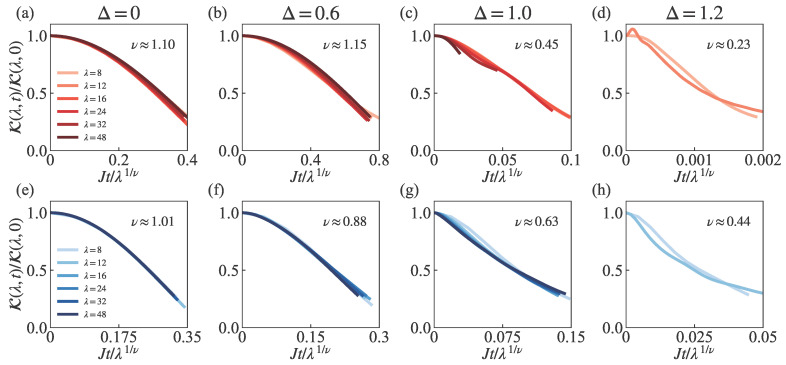
Scaling collapse analysis using the scaling exponents obtained from Figure 5 and Figure 6. Panels (**a**–**d**) correspond to the SH state, while panels (**e**–**h**) correspond to the MPDW state. Columns from left to right show results for Δ=0.0, 0.6, 1.0, and 1.2. After rescaling the evolution time Jt by $\lambda^{1/\nu }$, the curves with colors ranging from light to dark represent wavelengths λ=8,12,16,24,32,48. All normalized contrast curves for different wavelengths collapse onto a single curve. The data shown cover the time range before the normalized contrast decays to the threshold of 0.3.

Figure 7 presents a systematic scaling collapse analysis validating the universal dynamic scaling. Using the exponents obtained in Figure 5 and Figure 6, we rescale evolution time Jt by $\lambda^{1/\nu }$ for both the SH state (a–d) and MPDW state (e–h) across four representative anisotropy values: Δ=0.0, 0.6, 1.0, and 1.2. The analysis includes data before the normalized contrast decays to the threshold of 0.3, covering an extended time evolution range up to Jt≈100 as demonstrated by our normalized contrast decay curves. Despite quantum fluctuations, all decay curves collapse onto a single curve, thereby confirming the robustness of our scaling relation $\tau ∼\lambda^{1/\nu }$. This collapse further demonstrates distinct dynamical scaling behavior for different initial states and anisotropic parameters in the XXZ spin chain.

## 4. Conclusions

Based on the comprehensive contrast analysis in this work, we summarize the key findings regarding spin transport in the quantum XXZ spin chain.

By introducing the exotic MPDW and SH states with periodicity, we have systematically characterized the scaling behavior of spin transport across different anisotropic points. Our analysis reveals that the relative contrast decay time at different periodicities shows identical scaling behavior compared with the transferred current, which provides direct guidance for experiments on multi-period states. Additionally, our results demonstrate that the scaling exponents ν vary significantly with the anisotropy Δ, and they exhibit distinct behaviors for the SH and MPDW states. In particular, we observe a continuous decrease in the scaling exponent with increasing Δ, which eventually enters the subdiffusive regime. This behavior highlights the interplay between anisotropic interaction strength and initial conditions in determining transport properties.

The distinct symmetries of the two states within the single-fermion-particle framework indicate that their evolution is governed by different protective symmetries. This universality suggests an underlying common mechanism governing nonequilibrium spin dynamics, which is independent of specific periodic structures.

These findings not only deepen our understanding of quantum transport in integrable systems but also provide insights into the role of initial conditions in nonequilibrium quantum statistics. The approach developed here offers a general framework for studying spin transport in quantum many-body systems. Future work may extend this method to other models and explore connections to hydrodynamics and fluctuation phenomena. Furthermore, our findings can be directly measured in current ultracold-atom experiments, where single-site resolution now enables a direct measurement of spin polarization. These efforts can pave the way for a more comprehensive understanding of nonequilibrium quantum systems, thereby advancing our understanding of quantum transport beyond linear responses.

## Data Availability

The original contributions presented in this study are included in the article. Further inquiries can be directed to the corresponding authors.
